# The Ratio of Emergency Department Visits for ILI to Seroprevalence of 2009 Pandemic Influenza A (H1N1) Virus Infection, Florida, 2009

**DOI:** 10.1371/currents.outbreaks.44157f8d90cf9f8fafa04570e3a00cab

**Published:** 2014-06-30

**Authors:** Richard S. Hopkins, Aaron Kite-Powell, Kate Goodin, Janet J. Hamilton

**Affiliations:** Florida Department of Health, Tallahassee, Florida, USA; Florida Department of Health, Tallahassee, Florida, USA; Florida Department of Health, Tallahassee, Florida, USA; Florida Department of Health, Tallahassee, Florida, USA

**Keywords:** H1N1, Influenza, Pandemic, Surveillance

## Abstract

Background. A seroprevalence survey carried out in four counties in the Tampa Bay area of Florida provided an estimate of cumulative incidence of infection due to the 2009 influenza A (H1N1) as of the end of that year’s pandemic in the four counties from which seroprevalence data were obtained Methods. Excess emergency department (ED) visits for influenza-like illness (ILI) during the pandemic period (compared to four non-pandemic years) were estimated using the ESSENCE-FL syndromic surveillance system for the four-county area. Results. There were an estimated 44 infections for every ILI ED visit. Age-specific ratios rose from 19.7 to 1 for children aged <5 years to 143.8 to 1 for persons aged >64 years. Conclusions. These ratios provide a way to estimate cumulative incidence. These estimated ratios can be used in real time for planning and forecasting, when carrying out timely seroprevalence surveys is not practical. Syndromic surveillance data allow age and geographic breakdowns, including for children.

## Introduction

Reliable information about the cumulative incidence of influenza, available in near real time as the epidemic is unfolding, would be extremely helpful in assessing the likely near-term course of an epidemic or pandemic, and in supporting decision-making about the public health response. If the reproductive number R0 of the epidemic is known, it is possible to estimate the cumulative incidence of infection at which the epidemic will peak and start its descent. Such information could be used for example to inform decisions about whether to deploy or to phase out temporary treatment facilities. Ideally such information would be obtained from repeated seroprevalence surveys, but these are likely to be resource-intensive and slow, and cannot be performed at all unless a reliable laboratory test is available early in the epidemic to detect infection with the agent responsible.

During the 2009 Florida pandemic of illness due to the 2009 pandemic influenza A (H1N1) virus (pH1N1), the Florida Department of Health (FDOH) relied on emergency department (ED) visits for influenza-like illness (ILI) as an indicator of the impact of influenza by geographic area and age, but had no way to infer the weekly or cumulative number of influenza infections in the community from these data in real time. This project was undertaken to develop a method to use ED visits for ILI to estimate the number of influenza infections in the community, in a more timely way than by carrying out a serosurvey (which may in any case not be practical if appropriate laboratory tests are not available to detect infection with the pandemic strain.)

In late November and early December, 2009, the Centers for Disease Control and Prevention and the Florida Department of Health (DOH) collaborated on a serologic survey of antibodies to the 2009 influenza A/H1N1 virus, among both children and adults. The methods and results of that survey are described in detail in a previously published paper 1 . Briefly, the seroprevalence survey enrolled subjects from a four county region in central Florida -- Hillsborough, Manatee, Pasco, and Pinellas Counties, -- which include the Tampa Bay region and the cities of Tampa, St. Petersburg and Clearwater. Leftover sera were obtained from 657 adult blood donors over the four day period from November 30, 2009 to December 3, 2009, and from 219 children seeking medical care for various conditions, over the 48 day period from November 14, 2009 to December 31, 2009. All personal identifiers were removed from the specimens before delivery to DOH. Age, date of specimen collection, gender, and zip code were retained for all subjects. This project was deemed not to be human subjects research by the Florida Department of Health Institutional Review Board. Serologic results were adjusted to reflect estimated proportions of the population who might have received 2009 H1N1 vaccine before their blood specimen was obtained, and to reflect estimated sensitivity of the assay used to detect antibodies to the pandemic virus. The results used here reflect a hemagglutination inhibition assay titer of ≥40.

As judged by multiple surveillance systems available to the Florida Department of Health 2, the period when sera were obtained for the serosurvey coincided with the effective end of the pandemic in Florida. Very little H1N1 influenza activity was detected after the first week of December. The vaccine campaign was just getting started in Florida at the time of this serosurvey; the vast majority of doses administered were given after this time period. Thus the period when the sera were obtained was close to optimal to obtain a good estimate of cumulative immunity due to wild virus infection among Florida residents.

Data from Florida facilities participating in CDC’s National Respiratory and Enteric Virus Surveillance System (NRVESS) indicated that during the September-November pandemic period, as during the same period in other years, few infections due to respiratory viruses other than influenza were detected. The exception was the normal RSV season in central and southern Florida, which showed a steadily rising number of detections of Respiratory Syncyctial Virus (RSV) from late August through the end of December.

## Methods

Visits to emergency departments for ILI

In 2009, information about ED visits in Florida was available daily in an electronic format from 135 out of Florida’s 205 licensed acute care hospitals, which account for more than 70% of the state’s ED visit volume. They are included in Florida’s Electronic Surveillance System for the Early Notification of Community-Based Epidemics (ESSENCE-FL). For this analysis, historical ED visit data provided by facilities enrolled in ESSENCE-FL since 2009 have been included to more closely simulate the situation in which a jurisdiction has 100% coverage of emergency department visits in its syndromic surveillance system.

The Agency for Healthcare Administration (AHCA) maintains records of emergency department visits across the state of Florida, based on required reporting by licensed hospitals. An aggregated data set of all ED visits by facility, month of visit, and age group (<5 years, 5-17 years, 18-24 years, 25-49 years, 50-64 years, and >65 years) for May through December 2009 for hospitals in the 4-county project area was obtained from AHCA. These aggregated data were used to assess the coverage proportion of ESSENCE-FL within the four-county area. The ratios of AHCA aggregated visit counts by facility to the total ED visit data from ESSENCE-FL reporting facilities in the four counties by month and by age group were used to estimate the completeness of ESSENCE data compared to AHCA data, and to adjust ILI visit counts to account for ED visits not captured in ESSENCE-FL (Table 1). This completeness ranged from 74.2% for children under age 5 to 91.9% for persons aged 18 to 24 years, and was 85.2% overall.

Syndrome and sub-syndrome categories in ESSENCE-FL are based upon the text of the patient’s chief complaint. Records are binned into sub-syndromes using weighted keyword matching 3. The combinations of weights are logically equivalent to a decision algorithm based on these chief complaint text elements. The ILI syndrome is created by the combination of Influenza or (Fever and (Cough or Sore Throat)) sub-syndromes, so that patient records with chief complaints of fever and cough, fever and sore throat, influenza, flu, flulike, H1N1, swine flu, and avian flu, or combinations of these, are included. Records with chief complaints that include flu shot, stomach flu, vaccination, or immunization are excluded. The free-text terms H1N1 and swine flu were added to the ESSENCE-FL sub-syndrome definitions in April 2009 and remained in place throughout the rest of the period covered by this analysis.

ESSENCE-FL visit data for ILI were extracted by facility and by age group for Week 21 2009 through Week 44 2009 for patients who resided in one of the four project counties. Week 21, 2009, was the first week where public health laboratory data indicated that >50% of influenza confirmations were pH1N1. Week 44, 2009, was 14 days before median date of serum collection, allowing time for antibody development. In 2009, week 21 was the week ending May 30, and week 44 was the week ending November 7. Data were also obtained for weeks 21 through 44 of calendar years 2007, 2008, 2010 and 2011. Data are shown in the Figure as counts by week, with each year's MMWR weeks superimposed. This allows a visual assessment of the year-to-year similarity of these data in the four non-pandemic years, and the striking magnitude and timing of ILI visits during the pandemic period of 2009.

**Figure:**
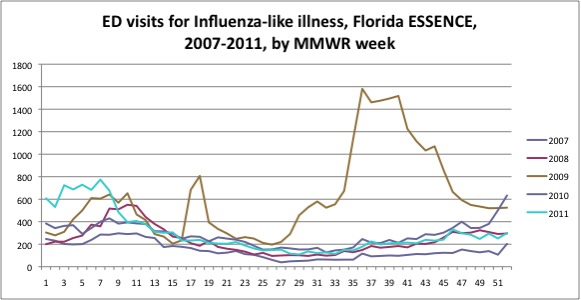

For each week from 21 to 44 in each of these five years, for each of the six age groups used in this analysis, the percentage of ED visits assigned to the ILI syndrome was ascertained. (Percentages were used rather than absolute numbers of ED visits for ILI, as the number of ESSNCE-FL participating hospitals was steadily rising during the period 2007-2009. Data before 2007 were and are too sparse to use as part of the baseline.) These percentages for the years 2007, 2008, 2010, and 2011 were averaged to obtain a baseline to which the pandemic year could be compared. For each week and age group, the excess ILI visit % was calculated by subtracting this baseline value from the 2009 value. For each week and age group, the excess ILI visit % was then expressed as a percentage of the 2009 percent ILI, to obtain the percentage of ILI visits that were excess visits. The actual number of 2009 ED visits for ILI, by week by age group, was multiplied by this percentage to obtain an estimated excess number of visits (compared to the expected number). Finally, all the excess ILI counts by week for each age group were summed to obtain an estimated number of excess ED visits for ILI by age group for the pandemic period from week 21 to week 44.

This analysis is based on routinely collected public health surveillance data, collected by the Florida Department of Health as authorized by law. Personal identifiers are not accessible to Florida Department of Health staff in ESSENCE-FL. Activities carried out as part of the Department’s surveillance function are not submitted to the Department’s Institutional Review Board for approval.

Estimates of Infected Individuals

The proportion of people infected with pH1N1 by age group was determined based on serology results from the CDC’s Tampa Bay area seroprevalence survey 1. Serologic results were adjusted to reflect estimated proportions of the population who would have received 2009 pH1N1 vaccine before their blood specimen was obtained, and to reflect estimated sensitivity of the assay used to detect antibodies to the pandemic virus. The results used here reflect a hemagglutination inhibition assay titer of ≥ 1:40. This proportion was then multiplied by age-specific 2009 population estimates for the four counties obtained from Florida CHARTS 4 to provide an estimate of infected individuals within the four-county region.

## Results

Comparisons by age group for May through December 2009 showed a range for completeness of ESSENCE visit data of 74.2 to 91.9% and no trend over time (data not shown).

The Figure shows the number of ILI visits by week for the state of Florida for calendar years 2007, 2008, 2009, 2010, and 2011. Weeks 17 and 18 of 2009 immediately followed the announcement of the detection of a novel influenza virus in California and Mexico. During those two weeks, the vast majority of influenza isolates in the Florida Bureau of Laboratories, for the state as a whole and for our four-county Tampa Bay region, were for other viruses (seasonal A/H3N2, seasonal A/H1N1, and B). The proportion of influenza isolates that were pH1N1 first reached 50% in week 21.

**Figure:**
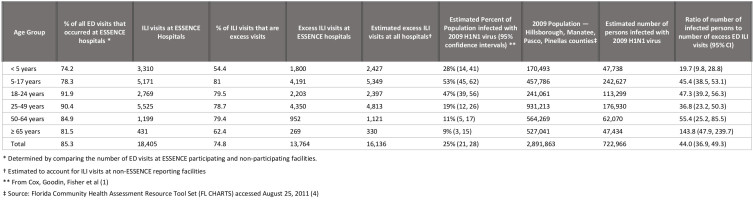
**Table 1. Estimation of Ratio of Number of Persons Infected with 2009 H1N1 Virus to Excess Number of Emergency Department Visits, in Pinellas, Hillsborough, Manatee and Pasco Counties, Autumn 2009.**

Applying the results of the seroprevalence survey to the four-county population (see Table), there were an estimated 722,966 persons infected with pH1N1 virus by early December. There were 18,405 ILI visits at Tampa Bay emergency departments from weeks 21 through 44, 2009. Of these, 13,764 (or 74.8%) were excess visits compared to our baseline from 2007, 2008, 2010 and 2011. Since 85.3% of all ED visits during May through December of 2009 were captured by ESSENCE, we estimated that there would have been a total of 13,764/0.853 or 16,136 excess visits in all Tampa Bay facilities. Similar calculations were done for each age group. Thus the estimated ratio of number of ED visits to number of people infected with pH1N1 was 16,136 to 722,966, or 1 to 44.0. This ratio varies from 1 to 19.7 in children aged 1 to 4 years, to 1 to 143.8 in persons aged 65 and over. The proportion of infections by age group is generally similar to the proportion of excess ED visits, with some variation. For example, 13.1% of excess ED visits were for children under age 5, while 9.7% of estimated infections were in that age group. For persons aged 65 years and older, 2.3% of excess ED visits and 6.5% of infections were in that age group.

## Discussion

During future influenza epidemics and pandemics, these estimates would allow the FDOH to make daily estimates of the cumulative number of infected persons with influenza by age group. Mathematical models indicate that the prevalence of immunity at which the epidemic will peak and start to decline is a function of the effective R_0, _the average number of people infected by each infected person 5. Being able to estimate cumulative population prevalence in real time thus provides information to those organizing the response to the epidemic, to address questions such as “How soon will the epidemic peak and start to decline” and “Is it necessary to plan for healthcare services for many more cases than we are seeing now?” Methods are available to estimate the effective R_0_ of an epidemic from prospectively collected influenza surveillance data 5, though they are subject to error, and to estimate the equilibrium seroprevalence using the R_0_ value 6.

During the fall of 2009, ED visit data suggested a disproportionate impact of this influenza epidemic on children aged less than 5 years old, with 18.0% of visits occurring in this group. Different decisions might have been made about priority groups for vaccine as it became available had it been known that only 13.1% of excess ED visits and 9.7% of infections were in this age group.

These estimates are subject to several limitations. As discussed in reference 1, estimated prevalence of antibodies due to wild virus infection from the serosurvey are subject to error. Future seasonal or pandemic influenza viruses may differ in average illness severity from that observed here, and thus the ratio of ED visits to infections may be higher or lower; and the distribution of severity by age may differ. Future versions of ESSENCE or other syndromic surveillance systems may change how they identify ED visits as being due to ILI, and thus reduce the generalizability of these results. Ratios based on cumulative infection percentages might not apply earlier in the pandemic. This method depends on having available stable baseline values of ED visits for ILI in non-pandemic years available, so that excess ILI visits may be estimated compared to expected values for each combination of week and age group.

During the pandemic period, essentially no other influenza viruses besides pH1N1 were being isolated in the Florida Bureau of Laboratories. Laboratories doing routine testing for RSV, and participating in the National Respiratory and Enteric Viruses Surveillance System (NREVSS) did show the expected seasonal increase in RSV detections during the period of the pH1N1 pandemic. When baseline ED visits for ILI from the non-pandemic years were compared to observed ILI visits for children under age 5, almost half of the total visits were accounted for by the baseline.

In a seasonal influenza epidemic, a high prevalence of partial pre-existing immunity to circulating viruses might alter the distribution of severity by age group compared to the 2009 pandemic, in which most people over age 65 had pre-existing immunity but younger people did not. This could limit the generalizability of these ratios during seasonal influenza.

In spite of these limitations, the FDOH plans to use the observed ratios between number of ED visits for influenza-like illness and number of infected persons, derived from this analysis, to assist in understanding the epidemiology of future outbreaks and pandemics of influenza. Similar analyses of data from other jurisdictions that carried out both a serosurvey and syndromic during the 2009 pandemic, or during other pandemics and during non-pandemic years, would help refine these estimates and help determine whether these estimated ratios are reproducible.

## Correspondence

Richard Hopkins: hopkinsrs@ufl.edu
